# Prognostic efficacy and prognostic factors of TACE plus TKI with ICIs for the treatment of unresectable hepatocellular carcinoma: A retrospective study

**DOI:** 10.3389/fonc.2022.1029951

**Published:** 2022-12-15

**Authors:** Ziqiang Han, Faji Yang, Ye Zhang, Jianlu Wang, Qingqiang Ni, Huaqiang Zhu, Xu Zhou, Hengjun Gao, Jun Lu

**Affiliations:** Department of Hepatobiliary Surgery, Shandong Provincial Hospital affiliated to Shandong First Medical University, Jinan, Shandong, China

**Keywords:** hepatocellular carcinoma, tyrosine kinase inhibitor, immune checkpoint inhibitor, transcatheter arterial chemoembolization, prognosis

## Abstract

Hepatocellular carcinoma (HCC) remains a global challenge due to its high morbidity and mortality rates as well as poor response to treatment. Local combined systemic therapy is widely used in the treatment of unresectable hepatocellular cancer (uHCC). This retrospective study was to investigate the prognostic effect and prognostic factors of transcatheter arterial chemoembolization (TACE) plus tyrosine kinase inhibitors (TKI) with immune checkpoint inhibitors (ICIs) in the treatment of uHCC. A retrospective analysis of 171 patients with uHCC was performed in our hospital from April 27, 2015 to October 18, 2021. According to different treatment options, patients were divided into TACE group (n=45), TACE+TKI group (n=76) and TACE+TKI+ICIs group (n=50). In this study, we found that, the median overall survival (mOS) of TACE+TKI+ICIs group was significantly better than TACE+TKI group and TACE group [24.1 (95% CI 15.1-33.1) months vs 14.9 (95% CI 10.7-19.1) months vs 11.4 (95% CI 8.4-14.5) months, hazard ratio (HR) 0.62; 95% CI 0.47-0.81; P=0.002]. A visible difference in the median progression-free survival (mPFS) interval between the groups was discovered [10.6 (95% CI6.5-14.7) months in TACE+TKI+ICIs group vs. 6.7 (95% CI 5.5-7.9) months in the TACE+TKI group vs. 6 (95% CI 2.3-9.7) months in the TACE group (HR 0.66; 95% CI 0.53-0.83; P<0.001)]. The objective response rates (ORR) in the TACE group, TACE+TKI group, and TACE+TKI+ICIs group were 31.1%, 35.5%, and 42%, and the disease control rate (DCR) were 51.1%, 65.8%, and 80%. There were no adverse events (AEs) of arthralgia, diarrhea, rash, and pruritus in the TACE group. The incidence of grade 3 AEs (Hypertension) in the TACE+TKI+ICIs group was significantly higher than that in TACE+TKI and TACE groups (28% vs 17.1% vs 6.7%, P=0.024), and secondly, the morbidity of rash and pruritus in the TACE+TKI+ICIs group was apparently higher than that in the TACE+TKI group (P<0.05). Multivariate analysis showed that ECOG-PS 2 (HR=2.064, 95%CI 1.335-3.191, P=0.001), Hepatitis B virus (HR=2.539, 95%CI 1.291-4.993, P=0.007), AFP≥400 ng/ml (HR= 1.72, 95%CI 1.12-2.643, P=0.013), neutrophil-lymphocyte ratio (NLR) ≥2.195 (HR=1.669, 95%CI 1.073-2.597, P=0.023) were independent risk factors for OS in uHCC patients. So, TACE+TKI+ICIs therapy can prolong the OS and improve the prognosis of patients effectively, with a well-characterized safety profile.

## Introduction

Primary liver cancer is the sixth most common cancer and the third leading cause of cancer deaths worldwide, with hepatocellular carcinoma (HCC) accounting for 75% to 85% (1). Radical therapy (partial hepatectomy, liver transplantation, or percutaneous radiofrequency ablation) provides good prognosis in patients with early-stage HCC (2). However, the pathogenesis of HCC is concealed, and most patients are in an advanced stage when diagnosed, with a low resection probability and bleak prognosis. What’s more, patients with large tumor burden, poor liver function, tumor thrombus in the portal vein or inferior vena cava, or extrahepatic metastasis are unable to receive radical treatment and the median overall survival (mOS) was 9 months. The main cause of death was tumor progression (3). With the exploration of the pathogenesis of HCC, the current treatment options for uHCC are transhepatic arterial chemoembolization (TACE), radiotherapy, systemic drug therapy, and so on.

The main blood supply to the normal liver parenchyma is the portal vein, while tumor tissue is supplied by the hepatic artery (4). Embolization after local chemotherapy can prolong the cytotoxic influence and reduce the systemic toxicity of chemotherapy (5). The dual blood supply from the hepatic artery and portal vein to the liver makes general arterial chemoembolization and arterial-directed therapy possible, and avoids normal liver parenchyma ischemia and hypoxia, reducing the damage of liver. So, TACE has a high application value in the treatment of hepatocellular carcinoma (6) (7), and is the most widely used treatment for uHCC (8, 9).

TACE interdicts tumor blood supply and causes tumor tissue ischemia. Residual tumor cells release hypoxia-inducible factor (HIF), leading to increased vascular endothelial growth factor (VEGF), fibroblast growth factor (FGF) and angiopoietin-2 (Ang-2), promotes tumor angiogenesis and progression, increasing the risk of cancer recurrence and metastasis (10). Tyrosine kinase inhibitors (TKIs), such as sorafenib, lenvatinib, apatinib, regorafenib and bevacizumab, inhibit the phosphorylation of tyrosine kinases. It can not only inhibit the proliferation of tumor cells by blocking the cell signaling pathway directly, but also inhibit the vascular endothelial growth factor receptor (VEGFR) and platelet-derived growth factor receptor (PDGFR) to block the formation of tumor angiogenesis indirectly (11, 12). Therefore, TACE plus TKI therapy has a synergistic effect in theory. Current studies have shown that TACE+TKI inhibits tumor progression and prolongs the survival of patients. For example, in the study of sorafenib with or without TACE, the median progression-free survival (mPFS) of sorafenib+TACE was significantly better than TACE (25.2 vs 13.5 months, P=0.006) (13).

Hepatitis induced by hepatitis B virus (HBV), hepatitis C virus (HCV), nonalcoholic fatty liver disease (NAFLD) or alcohol leads to inflammatory reaction and liver damage, is considered as the main cause of HCC pathogenesis (14). Therefore, HCC is considered an immunogenic tumor (15). T cells play an important role in the body’s anti-tumor immune activity, and the up-regulation of Programmed Death-Ligand 1(PD-L1) in tumor cells contributes to the immune suppressive microenvironment (16). Tumor immune checkpoint inhibitors (ICIs) are the most important aspect of tumor immunotherapy. By inhibiting the immune escape of tumor cells, the autoimmune system is mobilized to eliminate tumors. The research of tumor immune checkpoint inhibitors mainly focuses on three molecules: cytotoxic T-lymphocyte-associated protein 4 (CTLA-4), programmed cell death protein 1 (PD-1) and its ligand PD-L1, such as camrelizumab, sintilimab, pembrolizumab, atezolizumab, tislelizumab, nivolumab, ipilimumab. PD-1/PD-L1 immune checkpoint inhibitors have become a research hotspot in immunotherapy, and they have also achieved breakthrough progress in clinical practice.

TACE is targeted for the treatment of local tumors in the liver, and TKI and ICIs are used for systemic treatment through blood circulation to improve the systemic immune system. Combination therapy directly or indirectly inhibits local tumors, slows tumor growth, and even reduces tumor volume, providing the possibility of re-radical therapy for uHCC. However, whether combining ICIs with TKI plus TACE can improve survival in patients with uHCC remains unclear. Hence, this retrospective study aimed to evaluate the efficacy of TACE+TKI+ICIs on the survival of uHCC patients and the factors that affect their prognosis.

## Materials and methods

### Study design and patients

This study was approved by the Ethics Committee of the Shandong Provincial Hospital Affiliated to Shandong First Medical University (SWYX: NO.2022-191). The medical records of patients with unresectable HCC who were admitted to our hospital from June 8, 2016 to October 18, 2021 were retrospectively collected. Laboratory measurements within 3 days before TACE were collected from the hospital laboratory enquiry system. Ultimately, 171 patients were included in this study, and all met the following inclusion and exclusion criteria. The inclusion criteria comprised the following: 1. Histologically or cytologically confirmed HCC or clinically confirmed according to the criteria of the American Association for the Study of Liver Diseases; 2. Child-Pugh class A or B; 3. Physical performance status score ECOG-PS ≤ 2; 4. BCLC stages are B to C stages and A stage with contraindications to surgery (such as severe cardiopulmonary insufficiency);5. No prior TACE or systemic antitumor therapy for HCC;6.At least 1 measurable target nodule through the modified new criteria for tumor response evaluation (mRECIST, version 1.1); 7. Complete follow-up data. Exclusion criteria: 1. Past or current presence of other malignant tumors; 2. There are contraindications to TACE; 3. Child-Pugh class C; 4. Previous TACE or systemic therapy;5. Loss of follow-up data or incomplete patient data. The liver function classification standard adopts Child-Pugh classification of liver function. Child-pugh class A: 5-6 points; Child-pugh class B: 7-9; Child-pugh class C: ≥10 points. Tumors were staged according to the Barcelona stage (BCLC). The patient’s general condition, disease history, family history, preoperative blood routine, liver and kidney function, tumor markers, imaging and other report materials were collected through the case system.

### TACE therapy

The Seldinger method was used to place the 5F hepatic duct through the right femoral artery to the common hepatic artery. After the lesion was visualized by DSA, a microcatheter was inserted into the tumor feeding artery, and oxaliplatin and 5-FU were locally perfused. An appropriate amount of lipiodol mixed with epirubicin was then injected into the tumor lesions, and after the lipiodol was sufficiently deposited, DSA was performed again to evaluate the effect. TACE is performed as needed (patients undergo imaging examinations to evaluate the effect of lipiodol deposition in the lesions. If the deposition effect of lipiodol is good, TACE may not be performed temporarily; if the deposition of lipiodol subsides, TACE is required again.).

### TKI combined with ICIs therapy

TKIs are taken for a long time at doses after the first TACE, until the patient cannot tolerate the drug or the disease progresses, and then switch to other TKIs for subsequent treatment. ICIs were administered according to the applied dose on the first day after the first TACE and injected every 21 days thereafter. Medication use included in this article was the medication the patient was on at the end of follow-up. Doses of TKIs and ICIs (**Supplementary Table 1**).

### Follow-up and assessment

Follow-up-related data were obtained from patient follow-up phone calls, outpatient periodic review and readmission case data. The end of follow-up date was March 24, 2022. Overall survival (OS) definition: from the date of onset (or recurrence after liver resection) to the date of death or to the date of termination of follow-up, OS was the primary endpoint of this study. Progression-free survival (PFS) was defined as the period from the date of onset (or recurrence after liver resection) to the date of disease progression or death from any cause, whichever occurred first. Adverse events (AEs) were assessed according to the National Cancer Institute Adverse Events Common Terminology Criteria v 5.0. PFS and frequency of AEs were the secondary endpoints of this study. Efficacy was assessed according to m RECIST criteria: (1) Complete response (CR), the enhancement of intratumoral arteries in all target lesions disappeared or was completely inactivated; (2) Partial response (PR), the tumor survival residual cancer tissue (the sum of the diameters of all target lesions or enhancing lesions) is reduced by at least 30%; (3) Progressive disease (PD): tumor survival and residual cancer tissue increased by at least 20%, or new lesions appeared; (4) stable disease (SD): The tumor changes are between PD and PR.

### Statistical analysis

The collected data were systematically analyzed using SPPS version 26.0, and the categorical variables were expressed as percentages and analyzed by χ2 test. Survival curves were analyzed by Kaplan-Meier, and median overall survival (mOS) and mPFS were calculated, and graphs were drawn. The Receiver Operating Characteristic (ROC) curve was used to obtain the cutoff value of NLR for predicting OS. Cox regression proportional hazards model was used to perform univariate analysis on the screened clinical indicators, and multivariate analysis was performed on the statistically significant indicators in the univariate analysis to obtain independent risk factors for predicting tumor OS. Hazard ratios (HR) and 95% confidence intervals (CI) for these variables were estimated to quantify the strength of these associations. P<0.05 means there is a difference, which is statistically significant.

## Results

### Patient characteristics

This study retrospectively analyzed 294 uHCC patients in our hospital. According to the inclusion and exclusion criteria, 171 patients who met the criteria were finally included in this study. The patients were divided into TACE group, TACE+TKI group and TACE+TKI+ICIs group according to different treatment plans. The demographics and baseline characteristics were shown in **Table 1**. There were no significant differences in general clinical data such as gender, age, Eastern Cooperative Oncology Group performance status (ECOG-PS), Child-Pugh class, BCLC stage, portal vein tumor thrombus, tumor number, tumor size, HBV, AFP, ALT and NLR among the three groups of patients before treatment (P>0.05).

**Table 1:** Baseline data of patients included in the study.

Characteristics	TACE	TACE+TKI	TACE+TKI+ICIs	χ²value	P value
number	45	76	50		
Gender,n (%)				0.285	0.867
Male	38 (84.4)	65 (85.5)	41 (82.0)		
Female	7 (15.6)	11 (14.5)	9 (18.0)		
Age (years),n (%)				0.62	0.733
<60	26 (57.8)	42 (55.3)	25 (50.0)		
≥60	19 (42.2)	34 (44.7)	25 (50.0)		
ECOG-PS,n (%)				2.464	0.292
0-1	28 (62.2)	55 (72.4)	30 (60.0)		
2	17 (37.8)	21 (27.6)	20 (40.0)		
Child-Pugh class,n (%)				4.942	0.085
A	26 (57.8)	55 (72.4)	39 (78.0)		
B	19 (42.2)	21 (27.6)	11 (22.0)		
BCLC,n (%)				0.068	0.967
Stage A+B	30 (66.7)	49 (64.5)	33 (66.0)		
Stage C	15 (33.3)	27 (35.5)	17 (34.0)		
Portal vein tumor thrombus,n (%)				0.304	0.859
Yes	14 (31.1)	27 (35.5)	16 (32.0)		
No	31 (68.9)	49 (64.5)	34 (68.0)		
Tumor number,n (%)				4.99	0.083
Single	20 (44.4)	21 (27.6)	22 (44.0)		
Multiple	25 (55.6)	55 (72.4)	28 (56.0)		
Tumor size (cm),n (%)				0.924	0.63
<10	23 (51.1)	40 (52.6)	30 (60.0)		
≥10	22 (48.9)	36 (47.4)	20 (40.0)		
Hepatitis B virus,n (%)				3.447	0.178
+	35 (77.8)	68 (89.5)	44 (88.0)		
-	10 (22.2)	8 (10.5)	6 (12.0)		
AFP (ng/ml),n (%)				1.913	0.384
<400	22 (48.9)	33 (43.4)	28 (56.0)		
≥400	23 (51.1)	43 (56.6)	22 (44.0)		
ALT (U/L),n (%)				0.103	0.95
<40	22 (48.9)	35 (46.1)	24 (48.0)		
≥40	23 (51.1)	41 (53.9)	26 (52.0)		
NLR,n (%)				5.091	0.078
<2.195	14 (31.1)	32 (42.1)	27 (54.0)		
≥2.195	31 (68.9)	44 (57.9)	23 (46.0)		

TACE, transcatheter arterial chemoembolization; TKI, tyrosine kinase inhibitors; ICIs, immune checkpoint inhibitors; ECOG-PS, Eastern Cooperative Oncology Group performance status; BCLC, Barcelona Clinic Liver Cancer; AFP, alpha-fetoprotein; ALT, alamine aminotransferase; NLR, neutrophil-lymphocyte ratio

### Efficacy and safety

The DCR of TACE group, TACE+TKI group and TACE+TKI+ICIs group were 51.1%, 65.8% and 80%, respectively (P=0.012). The ORR in the TACE+TKI+ICIs group was 42%, while the ORRs in the TACE and TACE+TKI groups were 31.1% and 35.5% (P>0.05) (**Table 2**). AEs occurred in 166 (97%) patients. Among them, grade 1 and 2 AEs were in the majority, which could be well controlled after symptomatic treatment. Grade 3 AEs occurred in 119 patients, as shown in **Table 3**. The most frequent grade 3 AEs were elevated AST, elevated ALT, thrombocytopenia, hypertension, fatigue, fever, nausea, arthralgia, decreased appetite, diarrhea, pruritus, and rash. No grade 4 AEs occurred, and no patients experienced treatment-related deaths. Hypertension occurred in 3 patients in the TACE group, 13 patients in the TACE+TKI group, and 14 patients in the TACE+TKI+ICIs group (P=0.024). In addition, there were differences in pruritus (5.3% in the TACE+TKI group and 18% in the TACE+TKI+ICIs group, P=0.021) and rash (6.6% in the TACE+TKI group and 20% in the TACE+TKI+ICIs group, P=0.023), but no significant differences in other grade 3 AEs.

**Table 2:** Theraprutic efcacy of response.

Variable	TACE (n=45)	TACE+TKI (n=76)	TACE+TKI+ICIs (n=50)	P value
Best overall response,*n* (%)				
CR	1 (2.2%)	3 (3.9%)	3 (6%)	
PR	13 (28.9%)	24 (31.6%)	18 (36%)	
SD	9 (20%)	23 (30.3%)	19 (38%)	
PD	22 (48.9%)	26 (34.2%)	10 (20%)	
Objective response rate,*n* (%)	14 (31.1%)	27 (35.5%)	21 (42%)	0.536
Disease control rate,*n* (%)	23 (51.1%)	50 (65.8%)	40 (80%)	0.012

TACE, transcatheter arterial chemoembolization; TKI, tyrosine kinase inhibitors; ICIs, immune checkpoint inhibitors; CR, complete response; PR, partial response; SD, stable disease; PD, progressive disease.

**Table 3:** Key treatment-related adverse events of = grade 3.

Variable n %	TACE (n=45)	TACE+TKI (n=76)	TACE+TKI+ICIs (n=50)	P value
Elevated AST	8 (17.8)	15 (19.7)	15 (30)	0.281
Elevated ALT	8 (17.8)	14 (18.4)	11 (22)	0.844
Thrombocytopaenia	0 (0.0)	7 (9.2)	8 (16)	0.250
Hypertension	3 (6.7)	13 (17.1)	14 (28)	0.024
Fatigue	1 (2.2)	4 (5.3)	3 (6)	0.650
Fever	4 (8.9)	7 (9.2)	5 (10)	0.981
Nausea	7 (15.6)	11 (14.5)	7 (14)	0.976
Arthralgia	0 (0.0)	2 (2.6)	1 (2)	0.820
Decreased appetite	8 (17.8)	11 (14.5)	8 (16)	0.889
Diarrhea	0 (0.0)	5 (6.6)	6 (12)	0.464
Rash	0 (0.0)	5 (6.6)	10 (20)	0.023
Pruritus	0 (0.0)	4 (5.3)	9 (18)	0.021

TACE, transcatheter arterial chemoembolization; TKI, tyrosine kinase inhibitors; ICIs, immune checkpoint inhibitors; ALT, alamine aminotransferase; AST, aspartate aminotransferase.

### Survival analysis

**Figure 1:**
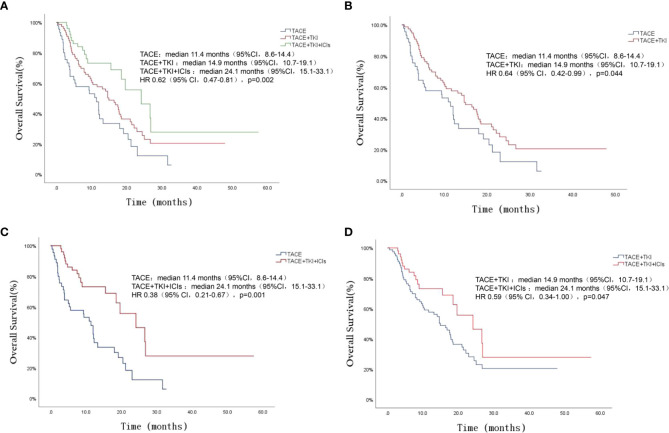
Kaplan-Meier survival curves showing OS stratified by treatment options in patients of uHCC. Comparison of OS among the three groups of patients **(A)**. OS comparison between TACE group and TACE+TKI group **(B)**. OS comparison between TACE group and TACE+TKI+ICIs group **(C)**. OS comparison between TACE+TKI group and TACE+TKI+ICIs group **(D)**.

**Figure 2:**
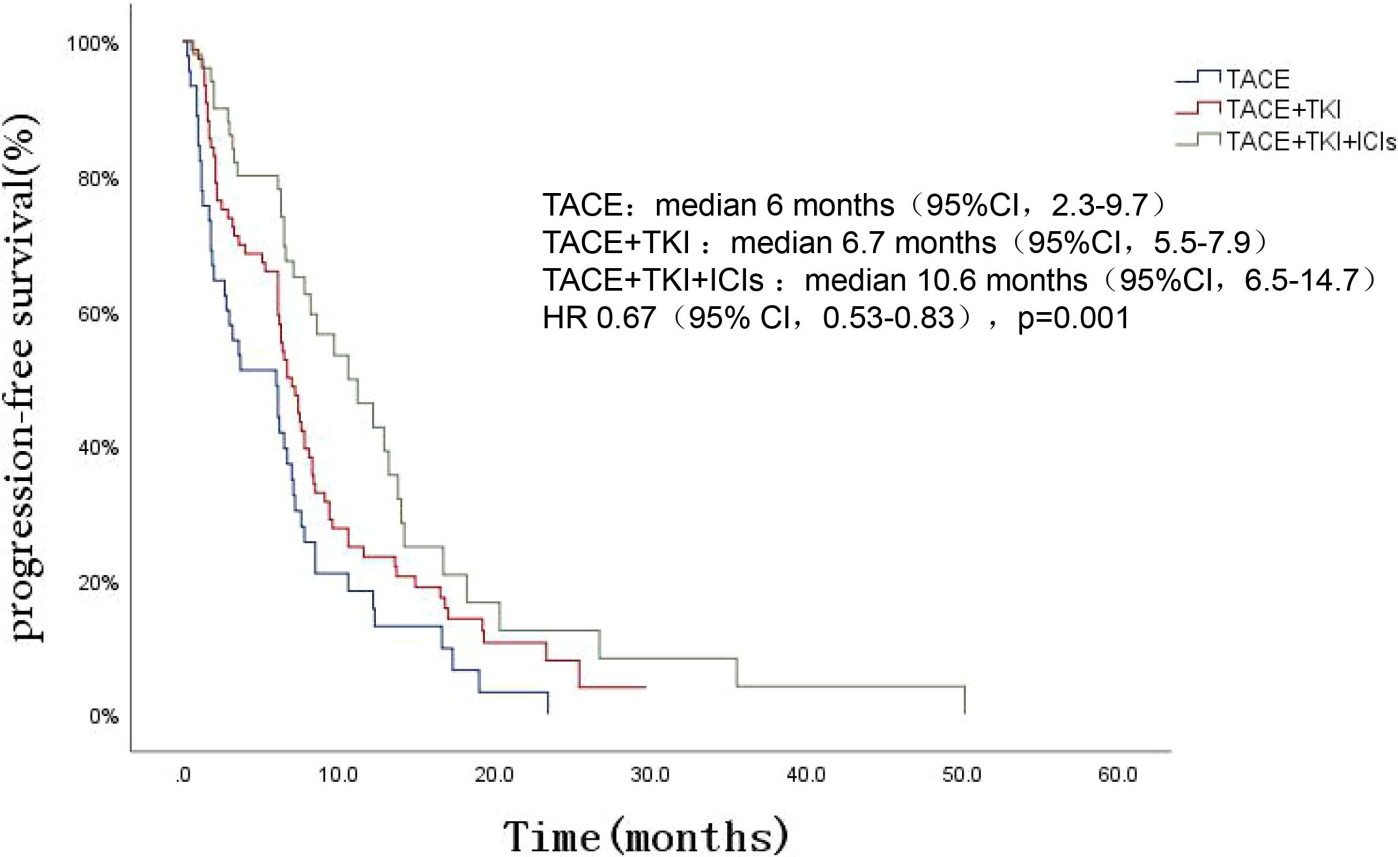
Kaplan-Meier survival curves showing PFS stratified by treatment options in patients of uHCC.

The patients’ survival of the three groups was followed up. The follow-up was from the date of TACE treatment to the date of death or termination of follow-up (March 2022). The total median follow-up time of the three groups was 15.5 (95%CI 11.8-19.2) months, among which the TACE+TKI+ICIs group had the longest mOS, followed by the TACE+TKI group, and the TACE group had the shortest mOS [mOS: 24.1 (95% CI 15.1-33.1) months vs 14.9 (95% CI 10.7-19.1) months vs 11.4 (95% CI 8.4-14.5) months, P=0.002], as shown in **Figure 1A**. And the TACE+TKI+ICIs group showed longer mOS than the TACE+TKI group (P=0.047, HR=0.585, 95%CI0.342-1.000), as shown in **Figure 1B**. Compared with the TACE group, mOS in the TACE+TKI+ICIs group and TACE+TKI group was prolonged by 12.7 months (P=0.001, **Figure 1C**) and 3.5 months (P=0.044, **Figure 1D**), respectively. So, compared with the TACE group and TACE+TKI group, the TACE+TKI+ICIs group had obvious advantages in prolonging the mOS of patients. Also, the TACE+TKI+ICIs group had the longest mPFS compared with the TACE and TACE+TKI groups, [mPFS: 10.6 (95% CI 6.5-14.7) months vs 6.7 (95% CI 5.5-7.9) months vs 6 (95%CI 2.3-9.7) months, P<0.001] (**Figure 2**). Also, there were differences among the treatment groups [(TACE vs TACE+TKI, P=0.043), (TACE vs TACE+TKI+ICIs, P<0.001), (TACE+TKI vs TACE+TKI+ICIs, P=0.042)]. Taken together, TACE+TKI+ICIs group significantly prolonged mPFS and mOS in uHCC patients.

### Risk factor analysis

**Figure 3:**
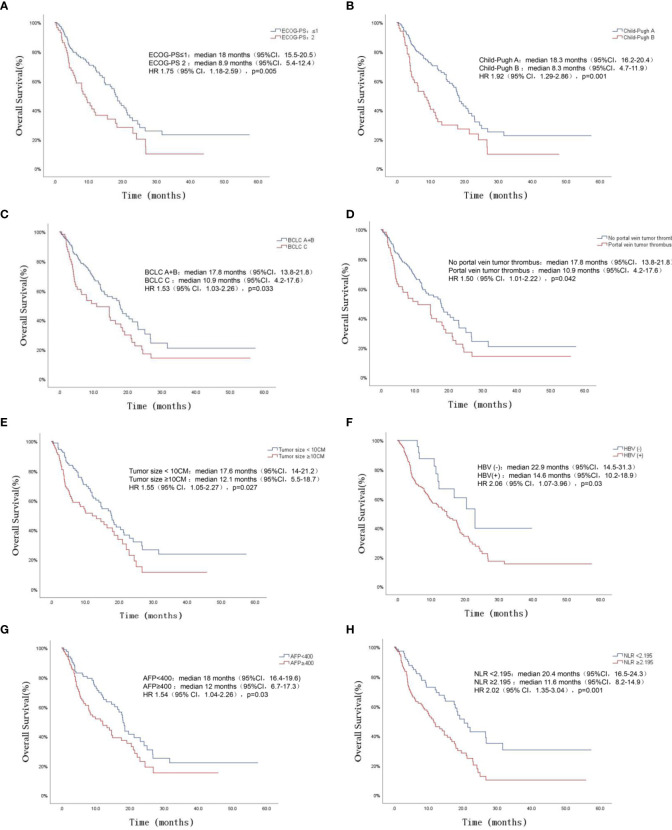
OS for patients of uHCC stratified by ECOG-PS **(A)**, Child-Pugh class **(B)**, BCLC staging **(C)**, Portal vein tumor thrombus **(D)**, Tumor size **(E)**, Hepatitis B virus **(F)**, AFP **(G)** and NLR **(H)** (all P < 0.05).

The cutoff value of NLR was obtained by the ROC curve, the largest of the NLR area under the curve (AUC) was 0.633, and the NLR cutoff value was 2.195 (P=0.003). Univariate analysis showed that ECOG score, Child-Pugh classification, BCLC stage, portal vein invasion, tumor size, HBV infection, AFP≥400, NLR ≥2.195 and different therapies were associated with OS of uHCC (P<0.05). The results of multivariate analysis showed: ECOG-PS (≤1vs2) (HR=2.064, 95%CI 1.335~3.191, P=0.001), HBV infection (yes vs no) (HR=2.539, 95%CI 1.291~4.993, P= 0.007), AFP (<400vs≥400) (HR=1.72, 95%CI 1.12~2.643, P=0.013), NLR (<2.195vs≥2.195) (HR=1.669, 95%CI 1.073~2.597, P=0.023) and different therapies (TACE vs TACE+TKI vs TACE+TKI+ICIs) (HR=0.544, 95%CI 0.402-0.736, P<0.001) were independent risk factors for OS in patients with uHCC, as presented (**Table 4**). Survival analysis showed that uHCC patients with ECOG-PS 2, Child-Pugh class B, BCLC stage C, portal vein tumor thrombus, tumor size ≥10 cm, HBV positive, AFP ≥ 400, and NLP ≥ 2.195 had a significantly shorter OS, as exhibited in **Figures 3A–H**.

**Table 4:** Analysis of OS prognostic factors in unresectable HCC patients.

Variable	N	mOS(month)	Univariate analysis			Multivariate analysis		
			P value	HR	95% CI	P value	HR	95% CI
Age(<60/≥60)	93/78	14.9/15.5	0.831	1.043	0.711~1.529			
Gender(Male/Female)	144/27	15.5/14	0.394	1.249	0.749~2.084			
ECOG-PS(≤1/2)	113/58	18/8.9	0.005	1.752	1.184~2.594	0.001	2.064	1.335~3.191
Child-Pugh class(A/B)	120/51	18.3/8.3	0.001	1.924	1.296~2.856			
BCLC(A+B/C)	112/59	17.8/10.9	0.033	1.527	1.034~2.257			
Portal vein tumor thrombus(Yes/No)	57/114	10.9/17.8	0.042	1.501	1.014~2.222			
Tumor number(Single/Multiple)	63/108	17.6/15.5	0.942	1.016	0.671~1.538			
Tumor size(<10/≥10)	93/78	17.6/12.1	0.027	1.546	1.052~2.271			
Hepatitis B virus(+/-)	147/24	14.6/22.9	0.03	2.062	1.074~3.958	0.007	2.539	1.291~4.993
AFP(<400/≥400)	83/88	12.0/18.0	0.03	1.535	1.042~2.259	0.013	1.72	1.12~2.643
ALT(<40/≥40)	81/90	17.6/14.6	0.337	0.91	0.751~1.103			
NLR(<2.195/≥2.195)	73/98	20.4/11.6	0.001	2.024	1.345~3.044	0.023	1.669	1.073~2.597
Treatment options(TACE/TACE+TKI/TACE+TKI+ICIs)	45/76/50	11.4/14.9/24.1	0.001	0.615	0.466~0.81	<0.001	0.544	0.402~0.736

TACE, transcatheter arterial chemoembolization; TKI, tyrosine kinase inhibitors; ICIs, immune checkpoint inhibitors; ECOG-PS, Eastern Cooperative Oncology Group performance status; BCLC, Barcelona Clinic Liver Cancer; AFP, alpha-fetoprotein; ALT, alamine aminotransferase; NLR, neutrophil-lymphocyte ratio; mOS, overall survival.

## Discussion

TACE treatment inhibites tumor growth through reducing tumor blood supply in uHCC and local infusion of chemotherapeutic drugs reduces systemic adverse reactions in patients. However, patients with uHCC treated with TACE alone suffered high possibility of tumor recurrence and metastasis, which induces short survival. Also, repeated TACE treatment seriously damages liver function and increases the risk of liver failure (17). TACE embolizes the liver blood supply, leading to hypoxis tumor microenvironment, releasing a large number of cytokines that promotes revascularization of the residual tumor tissue (18). Meanwhile, tumor hypoxic microenvironment leads to enhanced tumor cell invasiveness and promotes cancer metastasis (19). In this context, TACE plus TKI and ICIs make it possible. The combination therapy of TACE-TKI-ICIs can play a synergistic effect: 1. Reduce the blood supply of the local tumor, causing tumor ischemic necrosis; 2. Inhibit the vascular reconstruction of residual tumor tissue and reduce tumor metastasis and invasiveness; 3. Improve the level of autoimmunity and change the immune tolerance state of tumor microenvironment, as well as inhibit tumor immune escape. So, triple therapy can inhibit the progression of HCC and improve the prognosis and survival of patients with uHCC in theory. Also, in this study, we found that no grade 4 AEs occurred and no patients experienced treatment-related deaths. Hypertension, pruritus and rash occurred more frequently in triple therapy group, but no significant differences in other grade 3 AEs. So, triple therapy seems to be an effective and safe choice for patients with uHCC. However, greater sample sizes and a longer follow-up period are required to fully determine the long-term safety of triple therapy.

With the in-depth study of the tumor microenvironment (TME) and immune mechanism of liver cancer, the TME is involved in the occurrence, development, and metastasis of HCC. The TME plays a role in evading immune surveillance and promoting drug resistance tumor invasion, metastasis, resulting in poor efficacy (20). Therefore, combinatorial therapies will be the choice for uHCC (21). The main basis for the application of TKIs and ICIs is to adjust the TME from immune resistance to immune stimulation environment by anti-VEGF (22). Under such conditions, ICIs could promote anti-tumor immunity of T-cell (23). Recent study shows that low-dose apatinib modulates the tumor immunosuppressive microenvironment and enhances the anti-tumor effect of anti-PD-L1 medicine, which delays tumor growth, reduces the number of metastases, and prolonged survival in mouse models (24). In a phase Ib trial of lenvatinib combined with pembrolizumab in patients with uHCC, the mPFS and mOS of lenvatinib+pembrolizumab were 9.3 months and 22 months, respectively, reflecting the advantages of TKI+ICIs therapy for stable disease status (25). Another retrospective study showed the effect of lenvatinib+TACE and pembrolizumab+lenvatinib+TACE in the treatment of uHCC with PD−L1 expression. Pembrolizumab+lenvatinib+TACE treatment was more advantageous than lenvatinib+TACE in mOS and conversion rate [(18.1vs14.1, P=0.004), (25.7%vs11.1%, P=0.025)] (26). Also, TKI+ICIs treatment added the opportunity to for downstaging and resection of uHCC, (15.9% underwent R0 resection) (27); and another study showed the conversion rate was 60% after TACE+TKI+ICIs therapy (28). All the studies demonstrated that the combination of TKIs and ICIs was a feasible means of conversion therapy. A clinical study compared effect of first- and second-line treatment in advanced HCC with camrelizumab+ apatinib. In the first- and second-line groups, the ORR was 34.3% and 22.5%, the mPFS was 5.7 months and 5.5 months, and the one-year survival rates were 74.7% and 68.2%. So, the combination therapy strategy of TKI+ICIs can be used as a new choice for the first- and second-line treatment of HCC (29). A study of atezolizumab combined with bevacizumab versus sorafenib in the treatment of advanced uHCC (IMbrave150) showed that atezolizumab+bevacizumab significantly improved the patients’ mOS and mPFS compared with sorafenib [(19.2vs13.4, P<0.001), (6.9vs4.3, P<0.001)] (30). Also, the mPFS of sintilimab+bevacizumab was significantly longer than that of sorafenib (4.6vs2.8, P<0.0001), and the overall survival of the combination therapy was significantly better than that of sorafenib in the first overall survival analysis (ORIENT-32) (31). Except uHCC, TKI+ICIs treatment as preoperative neoadjuvant therapy for resectable HCC were also in development. Recent study showed that after preoperative neoadjuvant therapy with apatinib+camrelizumab, the pathological results of patients with HCC resection showed that 90% of the tumor resection tissue was necrotic and had no residual cancer cells, and the one-year recurrence-free survival (RFS) rate after HCC resection was 53.85% (32). A randomized phase 3 LEAP-012 study is ongoing: TACE with or without lenvatinib+pembrolizumab treatment of incurable intermediate-stage HCC was sesigned to verify the efficacy of triple therapy in prolonging OS and PFS (33). In this retrospective study, we found that TACE+TKI+ICIs treatment had obvious advantages in prolonging the survival time of uHCC compared with TACE and TACE+TKI. The mOS in the TACE+TKI+ICIs group was 24.1 months, and the mOS was prolonged by 12.7 months and 9.2 months compared with the TACE group and the TACE+TKI group. Compared with the TACE and TACE+TKI groups, the TACE+TKI+ICIs group had the longest mPFS [10.6 months vs 6.7 months vs 6 months, P<0.001]. So, for uHCC, TACE-TKI-ICIs triple therapy can effectively control the tumor progression, prolong the survival time of patients and improve the prognosis of patients.

Hepatocellular carcinoma is an inflammation-related cancer, mainly as a result of chronic liver damage or chronic inflammation (34). Long-term inflammatory stimulation causes liver fibrosis, which is an important component of the HCC TME. There are also a large number of vascular endothelial cells, immune cells (T cells, macrophages, neutrophils, dendritic cells) and cytokines in the TME (35). Virchow had already discovered a close relationship between inflammation and cancer in 1863 (36).Among them, serum NLR was considered as an index reflecting the inflammatory state. In this study, the high NLR suggested the worse the prognosis of patients., And the level of NLR may be closely related to the prognosis of advanced HCC. Accumulating evidence indicates that tumor-associated inflammatory response is closely related to the TME and plays an important role in cancer development, invasion and metastasis (37). Recent studies found that NLR above the threshold was closely related to shorter OS in head and neck squamous cell carcinoma, which was consistent with our data (38). Also, in other solid tumors, studies on the pre-correlation of NLR with its prognosis have also been carried out, and the results are consistent with the above study (39–41).

However, several limitations should be acknowledged. First, the types of TKIs and ICIs are not the same, there may be a selection bias, and it is impossible to accurately assess a certain triple therapy. Second, the causes of death in patients are less detailed, which has implications for overall survival analysis. Third, some imaging data of patients are from other hospitals, and the assessment of tumor status is biased. Fourth, this was a single-center study and the sample is small. So, multi-center and large-scale samples for further validation are needed. Fifth, the follow-up period is relatively short. Also, in this study, endpoint of follow-up was patient cannot tolerate the drug or the disease progresses followed with subsequent treatment. So, we didn’t refer patients received subsequent lines of therapy after the combination therapy of TACE+TKI+checkpoint inhibitors or OS between the cohorts - one with concurrent combination therapy versus sequential therapy. So, further research was need to explore whether there was a survival advantage for sequential therapy versus combination therapy. Finally, because kinds of TKIs or ICIs are not covered by medical insurance, the patients cannot afford multiple treatment costs, and there is a bias in the choice of drugs.

In conclusion, the results of this retrospective study show that compared with TACE alone and TACE+TKI, TACE+TKI+ICIs therapy has a better prognostic effect for uHCC.

## Data availability statement

The original contributions presented in the study are included in the article/**Supplementary material**. Further inquiries can be directed to the corresponding authors.

## Ethics statement

The studies involving human participants were reviewed and approved by Ethics Committee of the Shandong Provincial Hospital Affiliated to Shandong First Medical University. Written informed consent for participation was not required for this study in accordance with the national legislation and the institutional requirements.

## Author contributions

HG and JL. designed experiments. ZY, JW, QN and HZ. collected the data. ZH, FY and XZ analyzed and interpreted the data. ZH and FY wrote the manuscript. HG and JL made critical revisions to the article. All authors contributed to the article and approved the submitted version.

## References

[1] Sung H Ferlay J Siegel RL Laversanne M Soerjomataram I Jemal A. Global cancer statistics 2020: GLOBOCAN estimates of incidence and mortality worldwide for 36 cancers in 185 countries. CA Cancer J Clin (2021) 71(3):209–49. doi: 10.3322/caac.21660 33538338

[2] Forner A Reig M Bruix J. Hepatocellular carcinoma. Lancet (2018) 391(10127):1301–14. doi: 10.1016/s0140-6736(18)30010-2 29307467

[3] Giannini EG Farinati F Ciccarese F Pecorelli A Rapaccini GL Di Marco M. Prognosis of untreated hepatocellular carcinoma. Hepatology (2015) 61(1):184–90. doi: 10.1002/hep.27443 25234419

[4] Tsurusaki M Murakami T. Surgical and locoregional therapy of HCC: TACE. Liver Cancer (2015) 4(3):165–75. doi: 10.1159/000367739 PMC4608659 26675172

[5] Chang Y Jeong SW Young Jang J Jae Kim Y. Recent updates of transarterial chemoembolilzation in hepatocellular carcinoma. Int J Mol Sci (2020) 21(21). doi: 10.3390/ijms21218165 PMC7662786 33142892

[6] Llovet JM Real MI Montaña X Planas R Coll S Aponte J. Arterial embolisation or chemoembolisation versus symptomatic treatment in patients with unresectable hepatocellular carcinoma: A randomised controlled trial. Lancet (2002) 359(9319):1734–9. doi: 10.1016/s0140-6736(02)08649-x 12049862

[7] Han K Kim JH. Transarterial chemoembolization in hepatocellular carcinoma treatment: Barcelona clinic liver cancer staging system. World J Gastroenterol (2015) 21(36):10327–35. doi: 10.3748/wjg.v21.i36.10327 PMC4579879 26420959

[8] Takayasu K Arii S Ikai I Omata M Okita K Ichida T. Prospective cohort study of transarterial chemoembolization for unresectable hepatocellular carcinoma in 8510 patients. Gastroenterology (2006) 131(2):461–9. doi: 10.1053/j.gastro.2006.05.021 16890600

[9] Bruix J Sherman M. Management of hepatocellular carcinoma: An update. Hepatology (2011) 53(3):1020–2. doi: 10.1002/hep.24199 PMC3084991 21374666

[10] Kudo M. A new treatment option for intermediate-stage hepatocellular carcinoma with high tumor burden: Initial lenvatinib therapy with subsequent selective TACE. Liver Cancer (2019) 8(5):299–311. doi: 10.1159/000502905 31768341 PMC6872999

[11] Cucarull B Tutusaus A Rider P Hernáez-Alsina T Cuño C García de Frutos P. Hepatocellular carcinoma: Molecular pathogenesis and therapeutic advances. Cancers (Basel) (2022) 14(3). doi: 10.3390/cancers14030621 PMC8833604 35158892

[12] Awosika J Sohal D. A narrative review of systemic treatment options for hepatocellular carcinoma: State of the art review. J Gastrointest Oncol (2022) 13(1):426–37. doi: 10.21037/jgo-21-274 PMC8899752 35284102

[13] Kudo M Ueshima K Ikeda M Torimura T Tanabe N Aikata H. Randomised, multicentre prospective trial of transarterial chemoembolisation (TACE) plus sorafenib as compared with TACE alone in patients with hepatocellular carcinoma: TACTICS trial. Gut (2020) 69(8):1492–501. doi: 10.1136/gutjnl-2019-318934 PMC7398460 31801872

[14] Zhang C Yang M. The emerging factors and treatment options for NAFLD-related hepatocellular carcinoma. Cancers (2021) 13(15). doi: 10.3390/cancers13153740 PMC8345138 34359642

[15] Leonardi GC Candido S Cervello M Nicolosi D Raiti F Travali S. The tumor microenvironment in hepatocellular carcinoma (review). Int J Oncol (2012) 40(6):1733–47. doi: 10.3892/ijo.2012.1408 22447316

[16] McCaw ZR Ludmir EB Kim DH Wei LJ. Further clinical interpretation and implications of KEYNOTE-048 findings. Lancet (2020) 396(10248):378–9. doi: 10.1016/s0140-6736(20)30904-1 32771102

[17] Yang X Lan T Zhong H Zhang Z Xie H Li Y. To systematically evaluate and analyze the efficacy and safety of transcatheter arterial chemoembolization (TACE) in the treatment of primary liver cancer. J Healthc Eng (2022) 2022:8223336. doi: 10.1155/2022/8223336 35356619 PMC8959991

[18] Fernández M Semela D Bruix J Colle I Pinzani M Bosch J. Angiogenesis in liver disease. J Hepatol (2009) 50(3):604–20. doi: 10.1016/j.jhep.2008.12.011 19157625

[19] Liu Z Tu K Wang Y Yao B Li Q Wang L. Hypoxia accelerates aggressiveness of hepatocellular carcinoma cells involving oxidative stress, epithelial-mesenchymal transition and non-canonical hedgehog signaling. Cell Physiol Biochem (2017) 44(5):1856–68. doi: 10.1159/000485821 29237157

[20] Neophytou CM Panagi M Stylianopoulos T Papageorgis P. The role of tumor microenvironment in cancer metastasis: Molecular mechanisms and therapeutic opportunities. Cancers (Basel) (2021) 13(9). doi: 10.3390/cancers13092053 PMC8122975 33922795

[21] Sas Z Cendrowicz E Weinhäuser I Rygiel TP. Tumor microenvironment of hepatocellular carcinoma: Challenges and opportunities for new treatment options. Int J Mol Sci (2022) 23(7). doi: 10.3390/ijms23073778 PMC8998420 35409139

[22] Hegde PS Wallin JJ Mancao C. Predictive markers of anti-VEGF and emerging role of angiogenesis inhibitors as immunotherapeutics. Semin Cancer Biol (2018) 52(Pt 2):117–24. doi: 10.1016/j.semcancer.2017.12.002 29229461

[23] Kudo M. Scientific rationale for combined immunotherapy with PD-1/PD-L1 antibodies and VEGF inhibitors in advanced hepatocellular carcinoma. Cancers (Basel) (2020) 12(5). doi: 10.3390/cancers12051089 PMC7281246 32349374

[24] Zhao S Ren S Jiang T Zhu B Li X Zhao C. Low-dose apatinib optimizes tumor microenvironment and potentiates antitumor effect of PD-1/PD-L1 blockade in lung cancer. Cancer Immunol Res (2019) 7(4):630–43. doi: 10.1158/2326-6066.Cir-17-0640 30755403

[25] Finn RS Ikeda M Zhu AX Sung MW Baron AD Kudo M. Phase ib study of lenvatinib plus pembrolizumab in patients with unresectable hepatocellular carcinoma. J Clin Oncol (2020) 38(26):2960–70. doi: 10.1200/jco.20.00808 PMC7479760 32716739

[26] Chen S Wu Z Shi F Mai Q Wang L Wang F. Lenvatinib plus TACE with or without pembrolizumab for the treatment of initially unresectable hepatocellular carcinoma harbouring PD-L1 expression: A retrospective study. J Cancer Res Clin Oncol (2021) 148(8):2115–25. doi: 10.1007/s00432-021-03767-4 PMC9293824 34453221

[27] Zhu XD Huang C Shen YH Ji Y Ge NL Qu XD. Downstaging and resection of initially unresectable hepatocellular carcinoma with tyrosine kinase inhibitor and anti-PD-1 antibody combinations. Liver Cancer (2021) 10(4):320–9. doi: 10.1159/000514313 PMC8339461 34414120

[28] Zhang J Zhang X Mu H Yu G Xing W Wang L. Surgical conversion for initially unresectable locally advanced hepatocellular carcinoma using a triple combination of angiogenesis inhibitors, anti-PD-1 antibodies, and hepatic arterial infusion chemotherapy: A retrospective study. Front Oncol (2021) 11:729764. doi: 10.3389/fonc.2021.729764 34868921 PMC8632765

[29] Xu J Shen J Gu S Zhang Y Wu L Wu J. Camrelizumab in combination with apatinib in patients with advanced hepatocellular carcinoma (RESCUE): A nonrandomized, open-label, phase II trial. Clin Cancer Res (2021) 27(4):1003–11. doi: 10.1158/1078-0432.Ccr-20-2571 33087333

[30] Cheng AL Qin S Ikeda M Galle PR Ducreux M Kim TY. Updated efficacy and safety data from IMbrave150: Atezolizumab plus bevacizumab vs. sorafenib for unresectable hepatocellular carcinoma. J Hepatol (2022) 76(4):862–73. doi: 10.1016/j.jhep.2021.11.030 34902530

[31] Ren Z Xu J Bai Y Xu A Cang S Du C. Sintilimab plus a bevacizumab biosimilar (IBI305) versus sorafenib in unresectable hepatocellular carcinoma (ORIENT-32): a randomised, open-label, phase 2-3 study. Lancet Oncol (2021) 22(7):977–90. doi: 10.1016/s1470-2045(21)00252-7 34143971

[32] Xia Y Tang W Qian X Li X Cheng F Wang K. Efficacy and safety of camrelizumab plus apatinib during the perioperative period in resectable hepatocellular carcinoma: a single-arm, open label, phase II clinical trial. J Immunother Cancer (2022) 10(4). doi: 10.1136/jitc-2022-004656 PMC8981365 35379737

[33] Llovet JM Vogel A Madoff DC Finn RS Ogasawara S Ren Z. Randomized phase 3 LEAP-012 study: Transarterial chemoembolization with or without lenvatinib plus pembrolizumab for intermediate-stage hepatocellular carcinoma not amenable to curative treatment. Cardiovasc Intervent Radiol (2022) 45(4):405–12. doi: 10.1007/s00270-021-03031-9 PMC8940827 35119481

[34] Yu LX Ling Y Wang HY. Role of nonresolving inflammation in hepatocellular carcinoma development and progression. NPJ Precis Oncol (2018) 2(1):6. doi: 10.1038/s41698-018-0048-z 29872724 PMC5871907

[35] Baghban R Roshangar L Jahanban-Esfahlan R Seidi K Ebrahimi-Kalan A Jaymand M. Tumor microenvironment complexity and therapeutic implications at a glance. Cell Commun Signal (2020) 18(1):59. doi: 10.1186/s12964-020-0530-4 32264958 PMC7140346

[36] Balkwill F Mantovani A. Inflammation and cancer: back to virchow? Lancet (2001) 357(9255):539–45. doi: 10.1016/s0140-6736(00)04046-0 11229684

[37] Mantovani A Allavena P Sica A Balkwill F. Cancer-related inflammation. Nature (2008) 454(7203):436–44. doi: 10.1038/nature07205 18650914

[38] Cho JK Kim MW Choi IS Moon UY Kim MJ Sohn I. Optimal cutoff of pretreatment neutrophil-to-lymphocyte ratio in head and neck cancer patients: A meta-analysis and validation study. BMC Cancer (2018) 18(1):969. doi: 10.1186/s12885-018-4876-6 30309318 PMC6182814

[39] Bowen RC Little NAB Harmer JR Ma J Mirabelli LG Roller KD. Neutrophil-to-lymphocyte ratio as prognostic indicator in gastrointestinal cancers: a systematic review and meta-analysis. Oncotarget (2017) 8(19):32171–89. doi: 10.18632/oncotarget.16291 PMC5458276 28418870

[40] Ethier JL Desautels D Templeton A Shah PS Amir E. Prognostic role of neutrophil-to-lymphocyte ratio in breast cancer: A systematic review and meta-analysis. Breast Cancer Res (2017) 19(1):2. doi: 10.1186/s13058-016-0794-1 28057046 PMC5217326

[41] Zhan H Ma JY Jian QC. Prognostic significance of pretreatment neutrophil-to-lymphocyte ratio in melanoma patients: A meta-analysis. Clin Chim Acta (2018) 484:136–40. doi: 10.1016/j.cca.2018.05.055 29856976

