# Supplementary material for: Prognostic efficacy and prognostic factors of TACE plus TKI with ICIs for the treatment of unresectable hepatocellular carcinoma: A retrospective study
Source: Front Oncol. 2022 Dec 15;12:1029951. doi: 10.3389/fonc.2022.1029951 (PMC9798199; doi:10.3389/fonc.2022.1029951)
Supplement: Supplementary file 1 [file Table_1.docx]

| **Supplementary Table 1** | | | |
| --- | --- | --- | --- |
|  |  |  |  |
|  |  |  |  |
| Types and doses of TKIs used in the two groups | | | |
| Type | Dose (mg) | TACE+TKI(n=76) | TACE+TKI+ICIs(n=50) |
| Sorafenib | 200mg bid | 47 | 14 |
| Apatinib | 850mg qd | 18 | 11 |
| Regorafenib | 160mg qd | 7 | 7 |
| Lenvatinib | 8mg(<60kg)、 12mg(≥60kg) qd | 4 | 18 |
|  |  |  |  |
|  |  |  |  |
|  |  |  |  |
| Types and doses of ICIs | | |  |
| Type | Dose (mg) | TACE+TKI+ICIs(n=50) |  |
| camrelizumab | 200 | 27 |  |
| sintilimab | 200 | 10 |  |
| tislelizumab | 200 | 12 |  |
| pembrolizumab | 200 | 1 |  |

Abbreviations: TACE, transcatheter arterial chemoembolization; TKI, tyrosine kinase inhibitors; ICIs, immune checkpoint inhibitors
